# Preliminary Development and Validation of a New End-of-Life Patient-Reported Outcome Measure Assessing the Ability of Patients to Finalise Their Affairs at the End of Life

**DOI:** 10.1371/journal.pone.0094316

**Published:** 2014-04-15

**Authors:** Nikki McCaffrey, Pawel Skuza, Katrina Breaden, Simon Eckermann, Janet Hardy, Sheila Oaten, Michael Briffa, David Currow

**Affiliations:** 1 Flinders Centre for Clinical Change and Health Care Research, Flinders University, Daw Park, South Australia, Australia; 2 2eResearch@Flinders, Central Library, Flinders University, Bedford Park, South Australia, Australia; 3 Discipline of Palliative and Supportive Services, Flinders University, Daw Park, South Australia, Australia; 4 Centre for Health Service Development, Australian Health Services Research Institute, University of Wollongong, Wollongong, New South Wales, Australia; 5 Department of Palliative and Supportive Care, Mater Health Services, Brisbane, Queensland, Australia; 6 Southern Adelaide Palliative Services, Repatriation General Hospital, Daw Park, South Australia, Australia; 7 Palliative Care Unit, Royal Adelaide Hospital, Adelaide, South Australia, Australia; Supportive care, Early DIagnosis and Advanced disease (SEDA) research group, United Kingdom

## Abstract

**Introduction:**

The ability of patients to finalise their affairs at the end of life is an often neglected aspect of quality of life (QOL) measurement in palliative care effectiveness research despite compelling evidence of the high value patients place on this domain.

**Objective:**

This paper describes the preliminary development and evaluation of a new, single-item, end-of-life patient-reported outcome measure (EOLPRO) designed to capture changes in the ability of patients to finalise their affairs at the end of life.

**Methods:**

Cognitive interviews with purposively sampled Australian palliative care patients (N = 9) were analysed thematically to explore content validity. Simultaneously, secondary analysis of data from a randomised controlled trial comparing ketamine and placebo for the management of cancer pain (N = 185) evaluated: construct validity; test-retest reliability; and responsiveness.

**Results:**

Preliminary findings suggest patients interpret the new measure consistently. The EOLPRO captures the ability to complete physical tasks and finalise practical matters although it is unclear whether emotional tasks or resolution of relationship issues are considered. Personal and financial affairs should be separated to allow for differences in ability for these two types of affairs. The significant correlation between performance status and EOLPRO scores (*r* = 0.41, p<0.01, n = 137) and expected relationships between EOLPRO and proximity to death and constipation demonstrated construct validity. Pre- and post-treatment EOLPRO scores moderately agreed (n = 14, κ = 0.52 [95% CI 0.19, 0.84]) supporting reliability. The measure’s apparent lack of sensitivity to discriminate between treatment responders and non-responders may be confounded.

**Conclusion:**

Based on the preliminary findings, the EOLPRO should be separated into ‘personal’ and ‘financial’ affairs with further testing suggested, particularly to verify coverage and responsiveness. Initial evaluation suggests that the single-item EOLPRO is a useful addition to QOL outcome measurement in palliative care effectiveness research because common palliative care specific QOL questionnaires do not include or explicitly capture this domain.

## Introduction

Despite compelling evidence that patients at the end of life and their informal carers highly value the ability to finalise affairs at the end of life, effectiveness studies rarely include or explicitly measure this domain. It’s over a decade since Steinhauser et al. [1], [2] reported 94% (320/340) of seriously ill American veterans rated having ‘financial affairs in order’ as very/important at the end of life. The ability to ‘complete things and prepare for life’s end’ was ‘very/extremely important’ in 87% (349/434) of older Canadian patients with advanced cancer and chronic end-stage medical disease [3] and ‘preparation’ was highlighted as an important issue to measure at the end of life during in-depth interviews with ten UK cancer patients [4]. Additionally, Patrick et al. [5] identified ‘preparation for death’ as a domain that should be included in a ‘quality of dying’ measurement tool based directly on feedback from focus groups (n = 47), one-to-one interviews (n = 52) and a review of the literature. Most recently, preparation for the end of life was identified as a key component of a ‘good death’ by 23 UK informants at different points along the dying trajectory [6]. Yet this important quality of life (QOL) domain is not routinely included in palliative care effectiveness research.

The European Organization for Research and Treatment of Cancer quality of life palliative care (EORTC QLQ-C15-PAL) [7], Functional Assessment of Chronic Illness Therapy - Palliative Care (FACIT-Pal) [8], McGill Quality of Life [9] and Memorial Symptom Assessment Scale questionnaires [10] do not include or explicitly capture the ability of patients to finalise their affairs at the end of life, yet these are amongst the most frequently used multidimensional instruments for measuring QOL in palliative care studies [11]. If outcome measures do not adequately highlight such domains, service provision may fail to address complex issues important to patients, like preparation for death, and focus solely on managing physical symptoms [12]. Hence, a new, single-item, end-of-life patient-reported outcome tool (EOLPRO) was developed to capture this domain whilst minimising any additional patient burden due to multiple assessments [13].

### Objective

The aims of this preliminary work were to develop and evaluate the relevant psychometric properties of the new single-item tool: content and construct validity; test-retest reliability for stability; and feasibility.

## Methods

The intention was to include the tool alongside several clinical and patient-reported outcomes in the Australian Palliative Care Clinical Studies Collaborative (PaCCSC) trials. PaCCSC is a multi-site research collaborative evaluating the net benefit of different palliative care pharmacological interventions in phase III studies [14]. Medications being studied, such as ketamine, could affect patients’ ability to finalise their affairs in preparation for death due to adverse effects, including sedation or confusion [15]. The initial PaCCSC studies administered the FACIT-Pal and EORTC QLQ-C15-PAL, neither of which includes the ability to finalise affairs. Consequently, a *single-item scale* measuring the ability of patients to finalise their affairs in preparation for death was sought to add to the other outcome measures, minimising additional respondent burden and promoting feasibility [16]. The most recent systematic review examining end-of-life measures [17] was used to identify single-item scales. Two measurement tools, the Palliative Care Outcome scale (POS) [18] and the Missoula VITAS quality of life index (MVQOLI) [19] met these criteria. However, the attribute-specific question in the MVQOLI has not been tested as an individual item and members of the PaCCSC Scientific Committee thought the item lacked clarity, whereas the POS question concerns specific rather than general practical matters and the measurement time frame is limited to three days (see Figure S1). Consequently, based on the MVQOLI and POS items, a new EOLPRO was constructed (see Table 1).

**Table 1:** The end-of-life patient-reported outcome measure.

	Not at all	A little bit	Some-what	Quite a bit	Very much
I am able to manage my personal and financial affairs as I would wish………	0	1	2	3	4

It is important to ensure that the new tool is psychometrically sound [20]. Consequently scale reliability and validity need to be assessed commensurate with the requirements of a single-item scale. The instrument should measure the concept it was designed to capture (content validity); have theoretically meaningful relationships with other measures (construct validity); and reproduce the same results in similar circumstances (test-retest reliability for stability) [21], [22]. Additionally, the measurement tool should pick up differences in actual observed outcomes when present (responsiveness) [20], [23] and should be appropriately designed for the target population (feasibility) [21].

The EOLPRO was evaluated in two ways: (1) investigation of the content validity and feasibility through cognitive interviews; and (2) psychometric evaluation using a subset of data from a randomised controlled trial (RCT) comparing subcutaneous ketamine and placebo for the management of cancer pain (Australian New Zealand Clinical Trials Registry 12607000501448) [24].

### 1. Content Validity and Feasibility

A qualitative study was conducted to investigate whether respondents interpret the measure as intended and key aspects of the domain of interest could be adequately represented and captured by the single-item scale (feasibility).

#### Settings and participants

Participants were recruited from patients under the care of the palliative care team at the Royal Adelaide Hospital (RAH), South Australia. Brief, semi-structured, face to face, cognitive interviews were conducted with participants to explore palliative care patients’ interpretation of the statement ‘I am able to manage my personal and financial affairs as I would wish’ and the associated response categories [25]. Participants were purposively sampled on age, gender, diagnosis (cancer, heart failure, chronic obstructive pulmonary disease (COPD), AIDS), education level and performance status, reflecting characteristics of the target population and covering a wide range of cases to detect variation [26]. The absolute sample size was determined by data saturation i.e., until new, dominant issues no longer emerged from the interviews [27]. Patients meeting the following criteria were eligible for the study: ≥18 years of age; advanced cancer or non-cancer life-limiting illness; knowledge of diagnosis and prognosis; physically and mentally competent; English-speaking; able to read the study questionnaire; cognitively intact, defined according to a Mini-Mental State Examination score [28] ≥19; and physically able to participate, defined as Australian-modified Karnofsky Performance Status (AKPS) [29] score ≥40. Ethical approval for the study (including the consent procedure) was gained through the Flinders University and Southern Adelaide Health Services Social and Behavioural Research Ethics Committee and the RAH Research Ethics Committee.

#### Data collection

**Figure 1:**
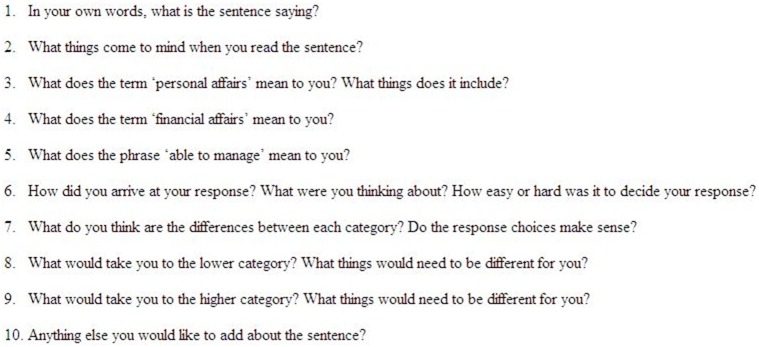
Scripted probes for the cognitive interview.

Written consent was obtained from participants. The signed consent form was inserted in the clinical file and a copy was given to the participant. Consented participants meeting the eligibility criteria took part in an interview with the lead author (NM) in their location of choice. First, the EORTC QLQ-C15-PAL questionnaire [7] was administered and completed individually, followed by the EOLPRO, reflecting questionnaire ordering in the PaCCSC studies. The EORTC QLQ-C15-PAL is an abbreviated 15-item version of the most widely used and validated cancer-specific HRQOL measure (the EORTC QLQ-C30 questionnaire), specifically developed for palliative care [7]. The questionnaire consists of 14 items, each with four possible responses (not at all, a little, quite a bit, and very much) and a QOL rating scale with seven categories ranging from 0 (very poor) to 7 (excellent). The participant’s interpretation of the EOLPRO was then explored and digitally recorded using scripted and spontaneous verbal probing (Figure 1). In verbal probing the interviewer asks specific questions about how the respondent answered a question with follow up probing if required. Verbal probing is thought to pose less of a burden as it requires less comprehension of thinking processes [30], is better at detecting ambiguity, and facilitates elicitation of specific types of information [26]. Consequently, brief, face to face cognitive interviews with verbal probing were chosen as the optimal approach for data collection for this frail population [26].

Recordings were transcribed verbatim and checked for accuracy. Willing participants underwent a second interview approximately five months later to comment on the accuracy of the analysis as a means of actively engaging the participants in the research process and verifying the results [31].

#### Data analysis

Demographic and clinical data were analysed using descriptive analysis. The transcribed interviews were analysed in QSR International’s NVivo version 8, 2008 (Doncaster, Australia) using constant comparative thematic analysis [32], [33]. Interview transcripts were iteratively reviewed and coded using open coding (data analysed using line-by-line coding), axial coding (data categorised and linked), and selective coding (overarching themes established and linked together) [32], [33]. Three of the interview transcripts were coded independently by a second researcher (KB) and the analysis was verified with members of the multi-disciplinary Palliative and Supportive Services Research Group at Flinders University [31]. Feedback interviews were analysed separately. The thematic analysis, and member and respondent verification informed: (i) the evaluation of the content validity of the EOLPRO; and (ii) the feasibility of measuring the ability to finalise affairs at the end of life with a single-item scale.

### 2. Psychometric Evaluation

Data from the multi-site, double-blind, parallel arm, dose titrated, Phase III PaCCSC RCT evaluating the net benefit of subcutaneous ketamine relative to placebo for the management of cancer pain (hereon in termed the ‘ketamine trial’) [24] were used to evaluate the psychometric properties of the EOLPRO.

#### Settings and participants

The total sample consisted of 185 inpatients with uncontrolled cancer pain despite aggressive use of standard analgesics [24]. Participants were randomised to titrated subcutaneous ketamine infusion or placebo for up to five days. The key eligibility criteria for the ketamine trial are summarised in Table 2.

**Table 2:** Key eligibility criteria in the ketamine trial [24].

Inclusion criteria	Exclusion criteria
Age >18 years	Previous ketamine use in the last six months
Pain related to cancer or its treatment	Unstable pain, or undergoing active treatment to reduce pain (surgery, chemotherapy, radiotherapy)
Moderate to severe pain	Medical history places patient at risk of known adverse reactions
Patients with either primarily nociceptive^a^ or predominantly neuropathic pain^b^ treated appropriately	Recent monoamine oxidase inhibitors
Stable background opioid dose	Previous recreational drug history
Stable co-analgesics during the study period	

a Leeds Assessment of Neuropathic Symptoms and Signs score (LANSS) <12;

b LANSS score >12.

#### Data collection

Measures in the ketamine trial relevant for the psychometric analysis included: the AKPS [29]; the Brief Pain Inventory scale (BPI) [34]; and the EORTC QLQ-C15-PAL questionnaire [7]. The AKPS is a validated measurement tool that assesses patient functioning and performance, and broadly correlates with prognosis in patients with cancer and AIDS [29]. The AKPS is an ordered, categorical scale with 11 levels and scores between 0 and 100; 0 represents death and 100 indicates normality, with no symptomatic complaints and no evidence of disease. The BPI is a numeric rating scale (0–10) which has been validated in advanced cancer and chronic pain [35]–[38]. The scale was used to measure the average pain severity where 0 represents ‘no pain’ and 10 indicates ‘pain as bad as you can imagine’ [39], [40].

#### Data analysis

Descriptive statistics were reported for demographic data and the measures listed above. Complete case analyses were conducted. All analyses were performed in PASW for Windows version 18 (SPSS Inc., Chicago, IL).

#### Construct validation

Construct validity was assessed by investigating hypothesised relationships between the EOLPRO scores and scores from the relevant, established scales in the ketamine trial using Spearman’s rank correlations [19]. Baseline EOLPRO scores were expected, at best, to moderately correlate (+) with baseline AKPS because the cognitive interviews suggested that a participant’s ability to manage their affairs is strongly influenced by their degree of independence, which is affected by physical and cognitive functioning. Lower levels of physical functioning were expected to reduce the ability to manage one’s affairs [41]–[44]. Baseline EOLPRO scores were hypothesises to weakly correlate (−) with BPI scores as greater levels of pain inhibit physical and cognitive functioning which indirectly influences the ability to manage one’s affairs [13], [40], [44], [45]. It was anticipated that baseline EOLPRO scores would moderately correlate (+) with proximity to death as the ability to manage one’s affairs at the end of life diminishes with declining physical and cognitive functioning as death approaches [46]–[48]. Finally, little or no correlation was anticipated between baseline EOLPRO scores and participants’ degree of constipation measured using the EORTC QLQ-C15-PAL question, ‘during the last week have you been constipated?’ (question 10) as this question measures an unrelated construct. Correlations of less than 0.3 were considered relatively weak, 0.30–0.50 moderate and >0.70 strong [16], [49], [50].

#### Test-retest reliability

Test-retest reliability for stability indicates whether a measurement tool produces consistent results when a condition is stable [51]. Establishing a ‘stable’ phase in a palliative care population is difficult given the different patterns of symptoms experienced by different patients, the expected continual functional decline over time and somewhat heterogenous trajectories before death [48]. Consequently, test-retest reliability was evaluated in a subgroup of the ketamine study participants, those with stable AKPS and BPI scores pre- and post-treatment. These participants were expected to have a stable clinical condition and stable ability to manage their affairs. Two definitions of stable scores were applied: equal pre- and post-treatment AKPS and pain scores; and equal pre- and post-treatment AKPS scores and post-treatment pain scores within plus or minus one category of the baseline pain score (as the minimal clinically important different pain score in the ketamine RCT was plus or minus two categories). Test-retest reliability was determined using the weighted Kappa Measure of Agreement which evaluates the degree of agreement between ordinal measures [51], [52]. A weighted kappa less than 0.2 was considered to indicate slight agreement, 0.21–0.40 fair, 0.41–0.60 moderate, 0.61–0.80 substantial and 0.81–1.00 almost perfect agreement [53]. Pre- and post-treatment EOLPRO scores were hypothesised to moderately, rather than substantially, agree because outcome measurements were taken five days apart and changes in other deteriorating symptoms such as fatigue, or treatment side effects, could affect the ability to manage affairs at the end of life in this subgroup of participants with stable pain [54], reducing test-retest agreement. Note the outcome measurement time points were pre-determined by the ketamine study protocol.

#### Responsiveness

Responsiveness of the EOLPRO was evaluated by investigating whether the measure discriminated between participants in the ketamine study who did and did not respond to treatment using the Chi-square test for independence [55]. Response was defined as: BPI average pain score at the start of Day 6 (i.e., after 5 days of ketamine/placebo) reduced by ≥2 points from baseline, in the absence of any increase in baseline opioid dose, and who had ≤4 breakthrough opioid doses in the last 24 hours; or a participant who withdrew before day 6, where the reason for withdrawal was unrelated to treatment and where the patient for whom a pain score at the start of day 6 was not available, but whose last recorded pain score was reduced ≥2 points from baseline and who had ≤4 breakthrough opioid doses in the last 24 hours [24]. Participants who responded to treatment were expected to experience an increased ability to manage their affairs compared with non-responders as pain levels influence levels of functioning [40]. Consequently, post-treatment EOLPRO scores were hypothesised to differ between responders and non-responders.

Results of the statistical tests were considered significant when the probability of making a type I error was less than 0.8% adjusting for multiple testing using the conservative Bonferroni method [56], [57].

## Results

### 1. Content Validation and Feasibility

Seventeen palliative care patients were invited to participate and nine patients consented. Reasons for not participating included: not stated (n = 3); not meeting the inclusion criteria (n = 2); lack of energy (n = 1); aversion to questionnaires (n = 1); and admission to hospice for terminal care prior to participation (n = 1). New, dominant issues no longer emerged by the ninth interview, i.e. data saturation was reached. Interviews lasted 6–23 minutes. Participant characteristics are summarised in Table 3.

**Table 3:** Qualitative study participant characteristics.

Characteristic	Total number (N = 9)
*Demographics*	
Age in years, mean (range)	69 (47–88)
Gender, M/F	3/6
English is the usual language spoken at home, n	9
Primary caregiver identified, n	7
Highest education level achieved, n	
Up to year 9	2
Years 10–12	4
Completed university or TAFE	2
Postgraduate	1
*Clinical data*	
Main life limiting illness, n	
Advanced cancer	4
Motor Neurone Disease	3
Heart Failure	1
COPD	1
Time since diagnosis (months), median (IQR)	12 (11)
min-max	1–120
MMSE score, mean (range)	28 (28–30)
AKPS score, n	
80	2
70	1
60	2
50	3
40	1

AKPS  =  Australian-modified Karnofsky Performance Status; COPD  =  chronic obstructive pulmonary disease; F  =  female; IQR  =  inter-quartile range; M  =  male; MMSE  =  Mini-Mental State Examination; SD  =  standard deviation; TAFE  =  Technical and Further Education.

There was a great degree of commonality between the participants’ interpretation of the phrase, ‘I am able to manage my personal and financial affairs as I would wish’; only one participant thought the question itself unclear. Participants wanted to manage their personal and financial affairs, and managing affairs helped them feel valued.

**Figure 2:**
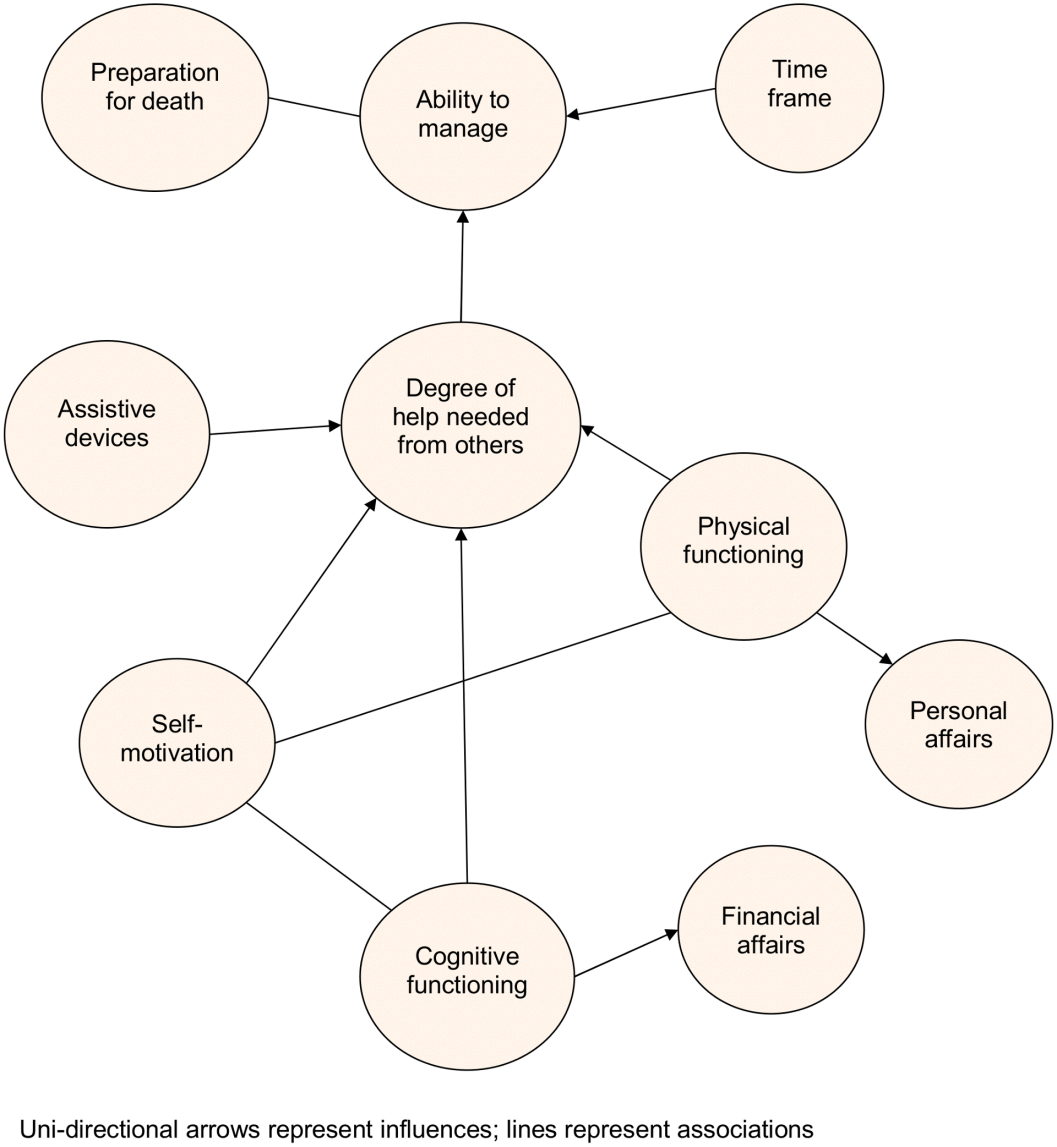
Links between key themes identified from the cognitive interviews.

Participants considered financial and practical matters (money, investments funeral arrangements, wills), personal care (hygiene, socialising, shopping) and preparation for death (handing over tasks to other family members, advance directives, saying goodbyes) when choosing a response category (see Figure 2). No-one explicitly discussed emotional tasks or resolving relationship issues.

The ability to complete activities without assistance from others ranked a higher EOLPRO score than those requiring assistance. Devices enabling interviewees to physically conduct activities, such as using a wheelchair for mobility or the computer to pay bills aided independence, promoting the ability to manage. Independence was strongly linked to cognitive and physical functioning. Cognitive abilities were most often tied to the ability to manage financial affairs whilst physical functioning was predominantly linked with personal affairs, with some overlap. Participants expected their physical and mental functioning would decline over time, reducing their ability to manage and participants wished to prepare for death before functional deterioration prevented them from completing activities such as arranging the funeral.

Most participants thought the categories were appropriate although two interviewees thought ‘very much’ meant that an individual could not manage their affairs. Nearly all of the participants suggested that the question should be split into two: one question about personal affairs and one about financial affairs. None of the participants (n = 4) reported inaccuracies in the thematic analysis during the follow-up interview. Three members of the research group verified ‘managing’, ‘personal and financial affairs’, and ‘expectation of abilities’ as major themes.

### 2. Psychometric Evaluation

The intention-to-treat study sample comprised 185 patients; four randomised participants withdrew before commencement of the study drug. Ketamine sample baseline characteristics are shown in Table 4. Demographically, the study population was fairly typical of the Australian cancer population [58].

**Table 4:** Ketamine sample baseline characteristics.

Characteristic		Number of cases (N = 185)
Age (years), mean (SD)	63.6 (11.9)	182
Gender, male (%)	103 (56.6)	182
Site of cancer diagnosis (n = 183), n (%)		
Lung	40 (21.9)	
Prostate	24 (13.1)	
Colorectal	22 (12.0)	
Breast	17 (9.3)	
Gynaecologic	11 (6.0)	
Pancreas	10 (5.5)	
Bone/soft tissue	7 (3.8)	
Other	52 (28.4)	
EOLPRO, median (IQR)^1^	3 (2)	137^2^
AKPS, median (IQR)^1^	60 (10)	182
EORTC QLQ-C15-PAL Question 10, median (IQR)^1^	2 (2)	160
BPI, mean (SD)^3^	5.3 (1.4)	181

1 the median and interquartile range are reported given the ordinal nature of the data;

2 the EOLPRO was introduced 5 months after study initiation;

3 the distribution of the BPI was approximately normal, hence the mean and standard deviation are reported; AKPS  =  Australian-modified Karnofsky Performance Status; BPI  =  Brief Pain Inventory Scale; EOLPRO  =  end of life patient reported outcome; IQR  =  interquartile range; SD  =  standard deviation.

Table 5 shows the proportion of missing values for the relevant, established scales in the sample data. The QOL measures had the greatest proportion of missing values.

**Table 5:** Proportion of missing values for key variables in the ketamine sample data.

Variable	Missing		Available	
	Baseline n (%)	Follow up n (%)	Baseline n (%)	Follow up n (%)
EOLPRO	48 (25.9)	81 (43.8)	137 (74.1)	104 (56.2)
EORTC QLQ-C15-PAL QOL	32 (17.3)	73 (39.5)	153 (82.7)	112 (60.5)
EORTC QLQ-C15-PAL Qu 10	25 (13.5)	71 (38.4)	160 (86.5)	114 (61.6)
AKPS	3 (1.6)	8 (4.3)	182 (98.4)	177 (95.7)
BPI score	4 (2.2)	13 (7.0)	181 (97.8)	172 (93.0)

AKPS  =  Australian-modified Karnofsky Performance Status; BPI  =  Brief Pain Inventory; EOLPRO  =  end of life patient reported outcome; MMSE  =  Mini-Mental State Examination; QOL  =  quality of life.

#### Construct validation

Data depicting construct validity are summarised in Table 6. As anticipated, there was a moderate, positive, statistically significant correlation between baseline AKPS and EOLPRO scores (*r* = 0.41, p<0.01), with greater performance status associated with an increased ability to manage affairs. Correlations between baseline EOLPRO scores and proximity to death (*r* = 0.30, p = 0.13) were moderate, positive and non-significant. In other words, longer survival was associated with an increased ability to manage affairs, as expected. Little correlation was found between baseline average pain and EOLPRO scores (*r* = 0.10, p = 0.25). Furthermore, this relationship was opposite to the anticipated direction. There was a negligible, non-significant correlation between baseline levels of constipation and EOLPRO scores (*r* = 0.02, p = 0.85), as anticipated, supporting divergent validity.

**Table 6:** Summary of the correlations between EOLPRO scores and other established scales and clinical measures.

Measure	Spearman’s correlation coefficient (95% CI)	Effect size	p-value^1^
*Convergent validity*			
AKPS (n = 137)	0.41 (0.26, 0.54)	moderate	<0.01*
BPI (n = 137)	0.10 (−0.08, 0.26)	very weak	0.25
Proximity to death (n = 28)	0.30 (−0.09, 0.59)	moderate	0.13
*Divergent validity*			
EORTC QLQ-C15-PAL Question 10 (n = 127)	0.02 (−0.15, 0.19)	negligible	0.85

1 correlations were considered significant when the probability of making a type I error was less than 0.8% to allow for multiple testing;

* statistically significant result; AKPS  =  Australian-modified Karnofsky Performance Status; BPI  =  Brief Pain Inventory; CI  =  bootstrap BCa confidence intervals.

#### Test-retest reliability for stability

The weighted Kappa Measure of Agreement suggested moderate agreement between pre- and post-treatment EOLPRO scores (stable definition 1, n = 14, κ = 0.52 (95% CI 0.19, 0.84) and stable definition 2, n = 32, κ = 0.48 (95% CI 0.25, 0.72) [53]. The 95% CIs were calculated using the web-based kappa with linear weighting calculator found at http://vassarstats.net/kappa.html.

#### Responsiveness

A Chi-square test for independence indicated no statistically significant difference in post-treatment EOLPRO scores between responders and non-responders (χ2 = 0.43, unadjusted p = 0.98; see Table 7). Furthermore, the Cramer’s V, suggested there was little, if any, association between responder status and post-treatment EOLPRO score (V = 0.06) [59].

**Table 7:** Post-treatment EOLPRO scores and responder status cross tabulation.

	Post-treatment EOLPRO scores n (%)					
	0: not at all	1: a little bit	2: somewhat	3: quite a bit	4: very much	Total
Responder	3 (8.6)	4 (11.4)	8 (22.9)	7 (20.0)	13 (37.1)	35
Non-responder	8 (11.6)	6 (8.7)	15 (21.7)	15 (21.7)	25 (36.2)	69
Total	11 (10.6)	10 (9.6)	23 (22.1)	22 (21.2)	38 (36.5)	104

## Discussion

The EOLPRO was developed to be used in addition to other palliative care QOL instruments to capture changes in the ability to manage one’s affairs in preparation for death for health services research. Very few QOL questionnaires consider constructs capturing this patient-valued domain. Within this context, the preliminary findings for content and construct validity, test-retest reliability, responsiveness and feasibility presented in this study are encouraging.

The thematic analysis, and member and respondent verification suggest that the EOLPRO adequately captures patients’ ability to complete physical tasks and finalise practical matters in preparation for death. Qualitative palliative care studies evaluating factors that are important to measure in the last weeks of life collectively suggest that ‘preparation’ should include: financial matters; funeral arrangements; writing a will; resolution of conflicts; emotional matters; completion of goodbyes; and legal arrangements [1], [2], [4], [5], [17], [18], [60]–[63]. Whilst virtually all of these items were mentioned during the cognitive interviews it is unclear whether the EOLPRO provokes thoughts of emotional and unresolved relationship issues or closure before death. Participants may have been unwilling to consider such painful aspects or to discuss personal and sensitive aspects of preparation for death. Such matters may not be relevant for individuals. Alternatively, the term ‘personal affairs’ may not resonate with participants who have not yet needed help with these aspects. Although the interview questions may have highlighted the ‘personal’ versus ‘financial’ issue (see questions 3 and 4, Figure 1), following the findings of the qualitative interviews, future iterations of the EOLPRO should split the statement into ‘personal affairs’ and ‘financial affairs’ to allow coverage of both aspects and improve content validity.

Although the sample size was relatively small and the interviews were short (median length 10 minutes) data saturation was reached by the ninth interview as new, *dominant* themes no longer emerged. For example, new facets of ‘preparation’ were no longer emerging by the ninth interview. Consequently it was considered unethical to continue interviewing more participants. The semi-structured cognitive interviews were kept deliberately brief due to the frail status of the population and focused on participants’ interpretation of the EOLPRO, particularly what activities were considered when thinking about personal and financial affairs and what ‘ability to manage’ meant. Also note the interview length does not include administration of the QOL questionnaires. There is evidence to suggest that six interviews with purposively sampled participants are sufficient to identify dominant issues [64]. Although one interview lasted only six minutes interpretation of the EOLPRO was adequately described with examples cited of personal and financial affairs, differences in the response categories outlined and consideration of response category detailed. This participant was an inpatient with an AKPS score of 50 (requires considerable assistance and frequent medical care).

The EOLPRO scores were moderately correlated with the AKPS scores providing support for convergent validity given the highly statistically significant and expected relationship between physical functioning and the ability to manage affairs at the end of life. Additionally, divergent validity is supported by the anticipated negligible relationship between baseline levels of constipation and EOLPRO scores.

The EOLPRO scores were only weakly correlated with pain scores, possibly due to the exclusion of patients with mild pain from the ketamine RCT [24]. Furthermore, the direction of the relationship between EOLPRO and pain scores was opposite to the anticipated direction. These unexpected results may be due to gender differences in pain scores. Although there was a negligible, negative, non-significant correlation between scores for males (*r* = −0.02, p = 0.87, n = 75), there was a weak, positive, statistically significant correlation for female participants (*r* = 0.27, p = 0.04, n = 62). Women ‘catastrophize’ pain more commonly than men [65], perhaps spurring end of life preparation. Alternatively, these results may be due to gender differences in perceptions of financial and personal matters, particularly as nearly all participants in the qualitative study suggested the EOLPRO should be split into one question about personal affairs and one about financial affairs. More work is required to understand the reported difference between genders and patients’ abilities to manage their affairs at the end of life.

When assessing stability, the results suggest, at best, a moderate agreement between the test-retest EOLPRO scores in participants with stable AKPS and average pain scores. Stable AKPS and average BPI scores were assumed to reflect clinical stability and therefore a stable ability to manage affairs. However, it cannot be excluded that pre- and post-treatment EOLPRO measures were captured under heterogenous conditions due to changes in other clinical symptoms, such as fatigue or breathlessness affecting the ability to manage affairs at the end of life [55], [66], leading to less than perfect test-retest agreement.

Response status may not have been significantly associated with EOLPRO scores as pain could be too indirectly related to the construct “preparation for death”, particularly given the very weak relationship between baseline EOLPRO and average BPI scores. Moreover, an interaction effect between gender and pain may be confounding the comparison. Further testing is required using more robust measures to support the responsiveness of the EOLPRO.

The new scale is designed to be used alongside multiple clinical and patient-reported outcomes in palliative care RCTs. Consequently, a single-item scale was chosen to capture the ability to manage affairs at the end of life in this frail population to minimise any additional administration and respondent burden associated with the new measure. Single-item scales tend to be simple and concise, easily interpreted, and quickly completed, whereas multi-item scales can be time consuming, difficult to interpret and burdensome [67], [68]. However, it may have been overly ambitious to hope one scale could cover all the key attributes of preparation for death [68]. Even though multi-item scales may improve coverage, consistency, stability, precision, reliability and responsiveness [67], [69] the practicality and feasibility [16] of the scale were considered important arguments against pursuing higher levels of psychometric acceptability typical in the development of rating scales in other clinical areas.

Approximately 47% of the baseline EOLPRO responses clustered in the ‘very much’ category suggesting nearly half of the sample could manage their affairs. A visual analogue scale (VAS) rather than a five category ordinal scale may have better captured variability in the domain, although empirical findings comparing the relative benefits of these scales are contradictory, and advantages are likely context specific [69], [70]. For example, as respondents get older and cognitive impairment increases, more errors have been reported when using the VAS compared with ordinal scales to measure pain intensity [71]. Categories in an ordinal scale may be easier for respondents to understand than a VAS anchored with extreme values [52], [71]–[73].

### Limitations

The qualitative and quantitative components of this study were conducted simultaneously as this preliminary work aimed to evaluate whether the single-item EOLPRO was fit for purpose rather than to develop the items for the scale. Conducting cognitive interviews before administration of the single-item scale could have usefully informed refinement of the measure to allow for differences in ability to finalise ‘personal’ or ‘financial’ affairs.

As with many longitudinal studies involving palliative care populations [74], [75] there is a sizeable proportion of missing values in the ketamine sample data. Palliative care patients’ health declines over time, fatigue may be more of an issue compared with other study populations, and outcome measurement can become burdensome, more readily leading to non-response or drop out. The missing values reduce the power to reject a false null hypothesis of no relationship between the chosen measures due to the smaller sample size from complete case analysis [76], particularly for weaker relationships. There was a higher proportion of missing data for the EOLPRO compared with the other QOL measures possibly due to outcome measure ordering as the EOLPRO was administered after the EORTC QLQ-C15-PAL and clinical measures. In a palliative care population, earlier administered outcome measures may be more likely to be completed given outcome measurement burden in this frail population. This finding further supports keeping measurement as simple as possible in a palliative care population.

The ketamine study population comprised solely of inpatients with chronic cancer pain who self-administered the EOLPRO after the EORTC QLQ-C15-PAL questionnaire. Validity, reliability and responsiveness of the EOLPRO can only be ascertained for similar administration conditions and patient populations.

## Conclusion

In conclusion, the EOLPRO is a single-item, end-of-life patient-reported outcome measure that was developed to capture changes in the ability of patients to finalise their affairs at the end of life whilst minimising any additional patient burden due to multiple assessments. The preliminary findings suggest the EOLPRO should be separated into ‘personal’ and ‘financial’ affairs with further testing suggested, particularly to verify coverage and responsiveness. Furthermore, implications of gender differences in patients’ abilities to manage their affairs and pain perception warrant additional investigation. Currently, common palliative care QOL questionnaires do not include or explicitly capture the ability of patients to finalise their affairs at the end of life despite compelling evidence that patients and their informal carers highly value this domain. Consequently, despite limited coverage, until an amended version of the single-item scale has been developed and tested, use of the single-item EOLPRO in addition to QOL outcome measurement is suggested as valuable in palliative care effectiveness research.

## Supporting Information

Figure S1 **The MVQOLI and POS attribute-specific questions.**
(DOC)
