# Supplementary material for: Preliminary Development and Validation of a New End-of-Life Patient-Reported Outcome Measure Assessing the Ability of Patients to Finalise Their Affairs at the End of Life
Source: PLoS One. 2014 Apr 15;9(4):e94316. doi: 10.1371/journal.pone.0094316 (PMC3988060; doi:10.1371/journal.pone.0094316)
Supplement: Figure S1 — The MVQOLI and POS attribute-specific questions. (DOC) [file pone.0094316.s001.doc]

The MVQOLI question [19]:

My affairs are in order;

I could die today with a clear mind

My affairs are not in order;

I am worried that many things are unresolved

The POS question [18]:

9. Over the past 3 days, have any practical matters resulting from your illness, either financial or personal, been addressed?

- Practical problems have been addressed and my affairs are as up to date as I would wish
- Practical problems are in the process of being addressed
- Practical problems exist which were not addressed
- I have had no practical problems
